# The Role of m^6^A on Female Reproduction and Fertility: From Gonad Development to Ovarian Aging

**DOI:** 10.3389/fcell.2022.884295

**Published:** 2022-05-30

**Authors:** Xiaoyan Sun, Jiafeng Lu, Hong Li, Boxian Huang

**Affiliations:** State Key Laboratory of Reproductive Medicine, Gusu School, Suzhou Municipal Hospital, Suzhou Affiliated Hospital of Nanjing Medical University, Nanjing Medical University, Suzhou, China

**Keywords:** m^6^A, ovary, oocyte, meiotic, reproduction aging

## Abstract

The growth and maturation of oocyte is accompanied by the accumulation of abundant RNAs and posttranscriptional regulation. N6-methyladenosine (m^6^A) is the most prevalent epigenetic modification in mRNA, and precisely regulates the RNA metabolism as well as gene expression in diverse physiological processes. Recent studies showed that m^6^A modification and regulators were essential for the process of ovarian development and its aberrant manifestation could result in ovarian aging. Moreover, the specific deficiency of m^6^A regulators caused oocyte maturation disorder and female infertility with defective meiotic initiation, subsequently the oocyte failed to undergo germinal vesicle breakdown and consequently lost the ability to resume meiosis by disrupting spindle organization as well as chromosome alignment. Accumulating evidence showed that dysregulated m^6^A modification contributed to ovarian diseases including polycystic ovarian syndrome (PCOS), primary ovarian insufficiency (POI), ovarian aging and other ovarian function disorders. However, the complex and subtle mechanism of m^6^A modification involved in female reproduction and fertility is still unknown. In this review, we have summarized the current findings of the RNA m^6^A modification and its regulators in ovarian life cycle and female ovarian diseases. And we also discussed the role and potential clinical application of the RNA m^6^A modification in promoting oocyte maturation and delaying the reproduction aging.

## 1 Introduction

RNA N6-methyladenosine (m^6^A) methylation was firstly reported in 1974 (Desrosiers et al., 1974; Perry and Kelley, 1974). Until 2011, the invention of m^6^A-specific methylated RNA immunoprecipitation with next-generation sequencing (MeRIP-seq) provided technical support for revealing the role of m^6^A methylation in eukaryotes (Dominissini et al., 2012; Meyer et al., 2012). m^6^A is the most prevalent internal modification in mRNA, non-coding RNA, ribosomal RNA, polyadenylated RNA (Krug et al., 1976; Rottman et al., 1976; Schibler et al., 1977; Fu et al., 2014; Meyer and Jaffrey, 2014), and it is composed of 0.1%–0.4% adenylate residues, most of which occurs on ‘RRACH’ (R = G or A; H = A, C or U) consensus sequence (Narayan and Rottman, 1988; Csepany et al., 1990). The m^6^A modification mainly enrich in the 5′- and 3′-untranslated regions (UTR), stop codons and last exon.

m^6^A modification has become an important branch of epigenetics independent of DNA methylation, histone modification, chromatin rearrangement and non-coding RNA regulation. m^6^A precisely regulated the RNA metabolism and gene expression including pre-mRNA processing, transport, localization, splicing, stability, degradation and translation of RNA (Wang et al., 2014; Wang et al., 2015; Xiao et al., 2016; He and He, 2021; Liu and Zhou, 2021), and played an important role in embryonic development, tumor occurrence, organ development and other post-transcriptional regulation in biological or pathological processes (Atlasi and Stunnenberg, 2017; Wang et al., 2018; Ma et al., 2019).

The m6A modifications are reversible and dynamically regulated by the m^6^A modulators. The formation of m^6^A is catalyzed by methyltransferase (METTL3/METTL14, WTAP, KIAA1429, RBM15 A/B, ZC3H13, and HAKAI) (Liu et al., 2014; Ping et al., 2014; Schwartz et al., 2014), and erased by demethylases (FTO and ALKBH5) (Jia et al., 2011; Zheng et al., 2013). m^6^A can be recognized by readers, such as YTH-domain family proteins (YTHDFs), YTH domain-containing proteins (YTHDC1/2), IGFBP1/2/3, HNRNPs, eIF3, LRPPRC, and Prrac2 (Xu et al., 2014; Liu et al., 2015; Du et al., 2016; Arguello et al., 2017; Patil et al., 2018; Yang et al., 2018; Wu et al., 2019; Wang et al., 2021a).

## 2 m^6^A Methylation Modification in Female Reproductive Development and Aging

### 2.1 m^6^A Methylation in Development and Aging

#### 2.1.1 m^6^A Methylation in Differentiations and Development

Many studies have confirmed that the differentiation and development of organs and tissues are precisely regulated by m^6^A modification accurately. For example, m^6^A played a decisive role on cell fate during the endothelial-to-haematopoietic transition to specify the earliest haematopoietic stem/progenitor cells during zebrafish embryogenesis, which was blocked in METTL3 deficient embryos (Zhang et al., 2017). The downregulation of ALKBH5 was responsible for the cardiomyocyte fate determination of human embryonic stem cells (hESCs) originated from mesoderm cells (Han et al., 2021). Currently, m^6^A modification in neuromuscular system development has been clarified in many studies. METTL3/METTL14, ALKBH5, FTO, YTHDF1/2/3 participated in the development, and disorders of nervous system (Li et al., 2018; Yu et al., 2021). The silence of FTO disturbed the skeletal muscle differentiation by suppressing mitochondria biogenesis and energy production involved in mTOR-PGC-1a pathway (Wang et al., 2017). m^6^A and its regulators participated in the proliferation and differentiation of myoblast, as well as muscle regeneration (Li J. et al., 2021).

In reproductive system, m^6^A methylation is involved in the entire process of spermatogenesis, including mitosis, meiosis, and spermiogenesis (Fang et al., 2021; Gui and Yuan, 2021). Knockdown of circGFRα1 mediated by METTL14 in female germline stem cells (FGSCs) significantly reduced their self-renewal (Li X. et al., 2021). Some enzymes were found to be involved in the oocyte development, such as METTL3 (Xia et al., 2018; Sui et al., 2020; Mu et al., 2021), YTHDC1 (Kasowitz et al., 2018), KIAA1429 (Hu et al., 2020) and YTHDF2 (Ivanova et al., 2017). However, there are few studies on m^6^A methylation modification about ovarian development.

#### 2.1.2 m^6^A Methylation in Aging/Senescence

Most studies showed m^6^A sites gradually increased with the aging process (Shafik et al., 2021). Abnormal m^6^A modification may be related to organ aging or cell senescence. Osteoporosis is a bone aging disease. m^6^A modification have been reported to be involved in regulating the proliferation, differentiation, and apoptosis of bone-related cells including bone marrow mesenchymal stem cells, osteoblasts, and osteoclasts by multiple studies (Huang et al., 2021) (Wang et al., 2021b; Chen et al., 2022) (Wu et al., 2020). In addition, IGF2BP2 highly expressed in Alzheimer’s patients by bioinformatic analysis using multiple RNA-seq datasets of Alzheimer’s brain tissues (Deng et al., 2021). Su et al. found that age difference impacted m^6^A RNA methylation in hearts and their response to acute myocardial ischemia/reperfusion (I/R) injury (Su et al., 2021). To date, increasing research have reported the connection between m^6^A and organ aging/cell senescence. However, most of them mainly focused on methyltransferase, such as METTL3/METTL14 (Zhang J. et al., 2020; Wu et al., 2020; Liu et al., 2021; Chen et al., 2022), and the demethylases and readers were gradually concerned in recent years, including ALKBH5 (Li et al., 2022a), WTAP (Li et al., 2022b), IGF2BP2 (Deng et al., 2021). Notedly, in reproductive aging, FTO has been shown to play the regulatory role (Ding et al., 2018; Zhou L. et al., 2021; Jiang et al., 2021; Sun et al., 2021). Meanwhile, m^6^A modification-related key downstream or upstream proteins have not been deeply studied.

### 2.2 The Role of m^6^A in Ovarian Development and Function

A few studies have been reported to prove that m^6^A played an important regulatory role in regulating ovarian development, ovarian function disorders and ovarian aging. In addition, m^6^A also affected the oocyte maturation, embryonic development, early organ formation and pregnancy process. Here, we mainly summarized current progress in the studies of m^6^A involved in oocyte development and maturation, ovarian function maintaining and highlighted its continuous and multiple influence in female reproduction and fertility.

Sexual differentiation began from 5 weeks after fertilization to 20 weeks in gestation. Primary sex cord (PSC) development from the gonadal ridge originated from the urogenital ridge and incorporated primordial germ cells (PGC) in XX genotype, which migrated into the gonad from the wall of the yolk sac. PSCs extended into the medulla and formed the rete ovary, which eventually deteriorated. The ovaries originally developed within the abdomen but later underwent a relative descent into the pelvis as a result of disproportionate growth (Dudex, 2013).

Recent studies showed that RNA methylation was involved in the ovarian development process. Sun et al. systematically analyzed the m^6^A level in ovaries, testes and detected the expression levels of several modification enzymes at different stages. They found that decreased demethylase (FTO and ALKBH5) and increased methyltransferase (METTL3 and METTL14) raised the m^6^A level during the development of gonads from 12.5 dpc as well as 7dpp to adult. In addition, m^6^A content was higher in luteal phase than follicular phase (Sun et al., 2020). Some regulators were uniquely and highly expressed in gonads. The expression of METTL3 was higher in ovaries and tests than other organs in Zebrafish (Xia et al., 2018). Moreover, the m^6^A regulated Sxl to facilitate sex determination in Drosophila (Kan et al., 2017). Currently, the mechanism of m^6^A is involved in gonadal development is not fully understood.

**FIGURE 1:**
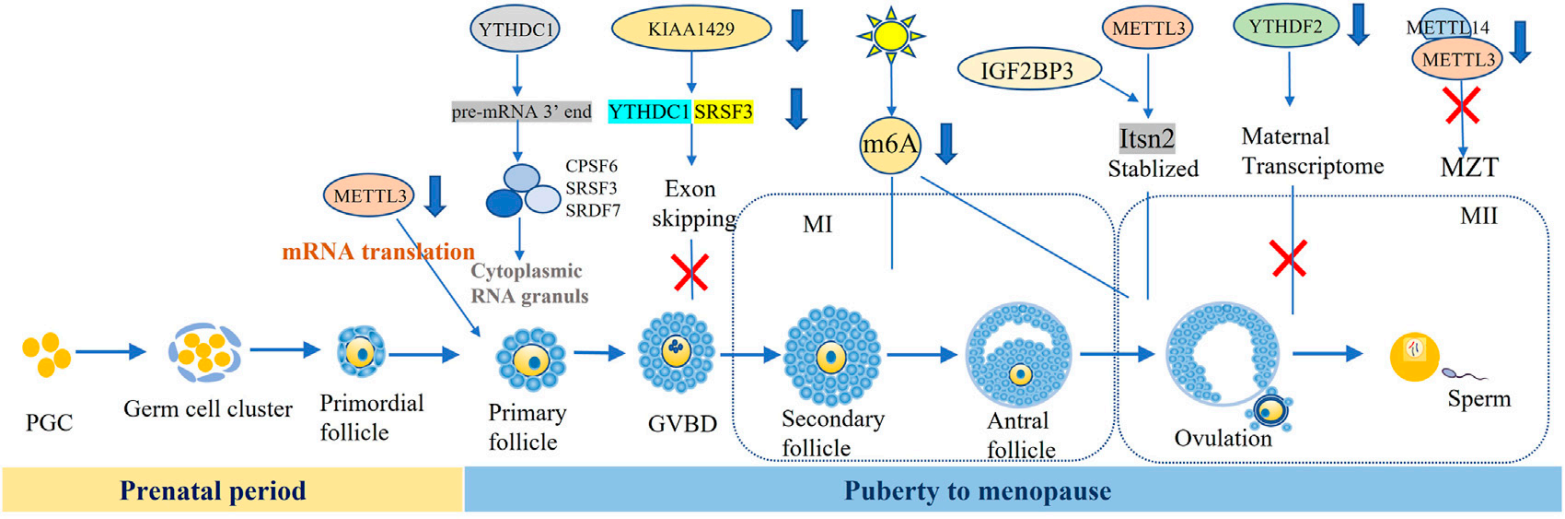
m^6^A and regulators participate in the oocyte development and matruration.

Ovarian aging is characterized by the constant decreasing of the number and the quality of follicles (Figure 1). The number of primordial follicles peaked at nearly seven million at gestational week 20 but dropped to one million at birth. At the menopausal age of 50 years old, about 1,000 follicles remained in ovary (Jerome and Robert, 2017; Laisk et al., 2019). Moreover, the more erroneous rate of meiosis during oocyte maturation increased with aging (Greaney et al., 2018). The aneuploidy rate of oocytes was 20% at the age of 35 and up to 60% at the age of 45 (Kuliev et al., 2011; Franasiak et al., 2014), resulting in the increasing incidence of aneuploid embryos, accompanied rising miscarriage, birth defection rate, gestational and obstetric complications (Hassold and Hunt, 2001; Frederiksen et al., 2018; Laisk et al., 2019; Correa-de-Araujo and Yoon, 2021). m^6^A modification and its regulators were essential for ovarian development and its aberrant manifestation resulted in ovarian aging. Jiang et al. found that downregulated FTO and increased m^6^A in granulosa cells (GCs) were accompanied by ovarian aging. They also reported that FTO slowed down the degradation of FOS-mRNA to upregulate FOS expression in GCs, eventually resulted in GC-mediated ovarian aging (Jiang et al., 2021). In 2009, FTO mutation was reported to cause severe growth retardation and accelerate senescence in the skin fibroblasts (Boissel et al., 2009). Meanwhile, Min et al. identified inconsistent results of m^6^A level during the aging process, which may be related to the tissue differences between the ovary and peripheral blood mononuclear cells (Min et al., 2018).

However, the expression pattern of m^6^A methyltransferase and reader in ovarian aging process have not been fully evaluated. Moreover, we don’t know the significance of m^6^A content and changes of its regulators as well as how to promote ovarian development. In the future, the significance of m^6^A and its regulators in the regulation of ovarian life cycle should be further studied.

### 2.3 m^6^A in Oocyte Development and Maturation

The process of oogenesis included three phases: growth, maturation, and ovulation. Maternal mRNA is activated in the early stage of oocytes and ceased at the germinal vesicle (GV) stage. The accumulated mRNA was pivotal for self-development of oocyte, post fertilization and early embryonic protein synthesis. Germinal vesicle breakdown (GVBD) was usually regarded as a hallmark of the progress of oocyte maturation. Then, the maternal mRNA began to degrade after LH peaks, and most polyadenylate mRNA disappeared before ovulation. At the two-cell stage, abundant maternal mRNA degraded. Sui et al. found that the signaling of m^6^A modification gradually decreased with degraded maternal RNAs in the cytoplasm from GV to two-cell stage. Conversely, this trend was opposite from two-cell to blastocyst stage (Sui et al., 2020).

mRNA from stable to unstable was an important step in oocyte cytoplasmic maturation and zygote transition. These indicated that post transcriptional regulation of mRNA methylation played a key role in oocyte maturation and zygote transformation. Here we summarized the roles of m^6^A modification in the oocyte developmental, such as follicle selection, meiosis maturation, and maternal zygote transformation (MZT).

#### 2.3.1 m^6^A Participated in the Follicular Selection

Although the role of m^6^A in mammalian follicular recruitment and selection has not been reported, there was a preliminary study on follicular selection by Hy-line Brown hens. MeRIP-seq data showed that chicken follicular transcriptome on average contained 1.61 and 1.59 m^6^A peaks per methylated transcript in pre-hierarchical and hierarchical follicles, respectively, which suggested that m^6^A methylation dynamic modification regulated chicken follicular selection (Fan et al., 2019). While the follicular recruitment and dominance in mammals remained to be studied.

#### 2.3.2 m^6^A Participated in the GV and GVBD Stages of Oocyte Development

Some researchers revealed that m^6^A methylation was involved in the arrest of GV stage oocyte, and METTL3 defection effected the development of GV oocyte. Recent study reported that METTL3 protein was indeed located in the oocyte nucleus at postnatal day (PD) 5 and 12, GV stage and granulosa cells. After knocking out METTL3 specifically in oocytes, the number of GV oocytes was significantly reduced, and the oocyte diameter of METTL3^cKO^ also became obviously smaller compared with WT mice. The deletion of METTL3 mainly effected the process of growing follicle development rather than the transition of primordial follicles to the activated growing follicles. Next, the researchers identified that METTL3/IGF3BP3-m^6^A-Itsn2 signaling axis participated in the oocyte development (Mu et al., 2021). In addition, the oocyte of *Ythdc1* ^*fl/-*^Ddx4-Cre ovaries was blocked at the primary stage, characterized by one layer of granulosa surrounding the oocytes, caused by the massive alternative splicing defects in YTHDC1 deficiency oocytes (Kasowitz et al., 2018).

The alteration of m^6^A methylation resulted in GVBD failure. Under the action of gonadotropin, oocytes underwent GVBD, that was the conversion of the prophase of the first meiosis to the first meiotic process (G_2_-M) (Zhou C. et al., 2021). METTL3 expressed at all stages of the oocyte and primarily distributed in the oocyte nuclei and granulosa cells. Most oocytes from METTL3^cKO^ mice could not undergo GVBD (Mu et al., 2021). Its homolog was identified as mettl3, Ime4, MT-A in Zebrafish, *Saccharomyces cerevisiae*, Arabidopsis thaliana (Xia et al., 2018)*.* Xia et al. reported that the arresting rate of primary growth stage (PG) oocytes was higher and full-grown (FG) stage follicles was significantly lower in Zmettl3m/m compared to WT respectively. The GVBD rate of these defective oocytes can be rescued by HCG and 17*α*-20*β*-DHP, which suggested that the competency of oocyte maturation was impaired by the mettl3 mutation (Xia et al., 2018). However, other study showed that GVBD rate was similar between GV oocyte microinjected with siRNA against METTL3, which suggested the meiosis resumption did not rely on METTL3 (Sui et al., 2020).

The KIAA1429-specific deficiency in oocytes resulted in female infertility with defective follicular development and GV oocytes failing to undergo GVBD, consequently losing the ability to resume meiosis. Loss of KIAA1429 could lead to abnormal RNA metabolism in GV oocytes by affecting the exon skipping events associated with oogenesis. KIAA1429 deletion caused the decreased localization of SRSF3 and YTHDC1 in the nucleus of oocytes, while enrichment of the SRSF3-binding consensus and YTHDC1-binding consensus were observed in the exon regions near the splicing sites (Hu et al., 2020).

At present, only some enzymes of m^6^A have been proved to be involved in GV and GVBD stages before meiosis, most of them have not been deeply studied. Therefore, the interaction between these adaptors and the factors related to oocytes development and the upstream and downstream target genes should be identified.

#### 2.3.3 m^6^A Participated in the Meiosis of Oocyte Maturation

Oocyte maturation was the committed process in sexual reproduction, referring to the process from the double line stage in the meiosis I (MI) to the meiosis II (MII). Various research reported m6A methylation were involved in the oocyte maturation process. METTL3 knockout in mammals and plants were embryonic lethality (Wang et al., 2014; Chen et al., 2015; Geula et al., 2015). Researchers microinjected the siRNAs against METTL3 to GV oocytes and found that the ratio of first polar body extrusion (PBE) was significantly decreased, although the similar GVBD rate implied no effect on meiotic resumption. In addition, obvious spindle abnormalities including elongated, wide-polar and short spindles were observed in about 50% of MII oocytes in METTL3 knockdown group (Sui et al., 2020). Recently, Mu et al. reported that MII oocytes were not produced in Mettl3 ^Gdf9−cKO^ mice, with only a small number of oocytes reached to the end of MI, and almost no first PBE occurring. Further, most of the oocytes from Mettl3^Gdf9−cKO^ mice could not be fertilized to form zygotes or develop beyond the four-cell embryo stage (Mu et al., 2021), which may affect the mRNA translation efficiency involved in chromosome congression and spindle formation due to METTL3 knockdown during oocyte maturation in mice. Another study also demonstrated that total translation efficiency of maternal mRNA was decreased in METTL3 knockdown oocytes (Sui et al., 2020). Moreover, environmental factors such as constant light exposure reduced the oocyte maturation rate by reduced m6A fluorescence intensity (Zhang H. et al., 2020).

Current research focused on the function of METTL3 in oocyte maturation, by affecting the translation efficiency, disrupting mRNA stability and expression of genes on sex hormone synthesis. Besides, other enzymes of m^6^A modification involved in meiotic of oocytes maturation have not been fully identified. We hope to map the integral mechanism network of m^6^A participating in oocyte development to help diagnose the causes of follicular development disorders and provide possible intervention ideas.

### 2.4 m^6^A Participated in Maternal-to-Zygotic Transition

MZT referred to that most maternal RNAs were gradually degraded after fertilization and the zygotic genome started to govern the gene expression, which phenomenon existed in all animal species (Zhao et al., 2017). Maternal mRNA clearance and the zygotic genome activation maintained early embryonic development. There was high 87% similarity in Zebrafish and human genes, so it was widely used as a model animal in the oocyte maturation and embryonic development in the research of m^6^A. YTHDF2 defection caused MZT failure by decelerating the decay of m^6^A-modified maternal mRNAs and disturbing zygotic genome activation (Zhao et al., 2017). Another study also showed that conditional mutagenesis of YTHDF2 disturbed maternal function in regulating transcript dosage of MII oocytes and further damaged early zygotic development in mice (Ivanova et al., 2017). Deng et al. reported that YTHDF2 was vital early embryogenesis as it advanced maternal mRNA clearance in goat (Deng et al., 2020). Another reader, IGF2BP2, was reported that maternal deletion of it caused early embryonic arrest at the 2-cell-stage in mouse embryos (Liu et al., 2019). In addition, METTL3 knockdown induced that the overall translation efficiency of maternal mRNA in oocytes was reduced, which further inhibited oocyte maturation and eventually impeded zygotic genome activation and MZT by disturbing the degradation of maternal mRNAs in MII oocytes (Sui et al., 2020).

## 3 m^6^A Modification Contributes to Ovarian Diseases

### 3.1 m^6^A and Ovarian Aging

Ovarian aging generally includes normal ovarian aging (NOA) and pathological ovarian aging (Zhang et al., 2019). NOA is defined as the gradual decline of oocyte quantity and quality with aging until menopause. Pathological ovarian aging refers to primary ovarian insufficiency (POI). In 2018, we firstly reported that m^6^A content in POI patients and CTX induced POI mice was significantly higher than normal groups, and the mRNA and protein expression levels of demethylase FTO were significantly lower in the POI patients than control group, which may be responsible for the increased risk of POI (Ding et al., 2018). Next, we identified that CTX raised the m^6^A and methyltransferase levels and inhibited the expression of demethylases and effectors with concentration-dependent (Huang B. et al., 2019). We also found that the expression of FTO reduced and m^6^A content was increased (Sun et al., 2021). Li team further verified that the downregulation of FTO increased m^6^A modification of FOS-mRNA-3′UTR and upregulated the expression of FOS in GCs, eventually resulting in the ovarian aging (Jiang et al., 2021). Zhu et at found that melatonin supplementation could protect the human ovarian surface epithelial cells to antagonize ovarian aging by suppressing the pathway of ROS-YTHDF2-MAPK-NF-κB (Zhu et al., 2022).

**FIGURE 2:**
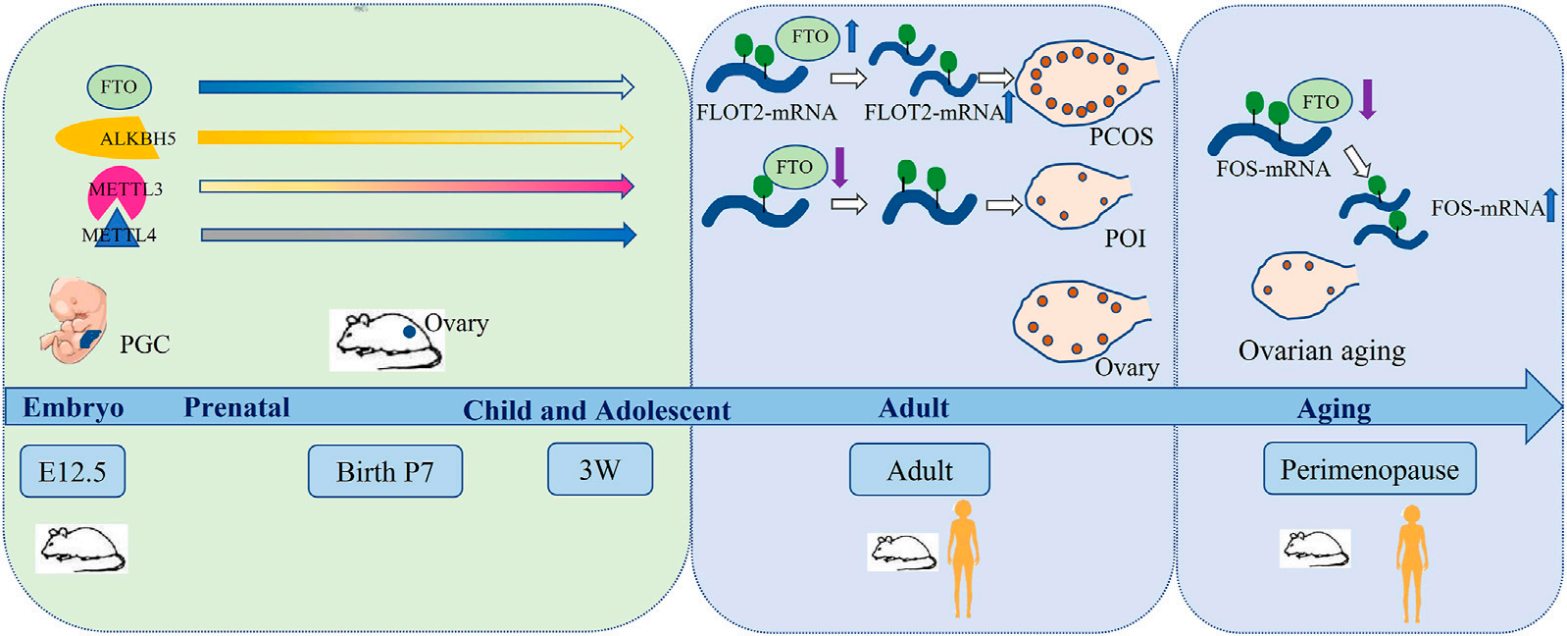
m^6^A and regulators participates in the ovarian life cycle.

The mechanism of ovarian aging and POI is involved in heredity, immunity, inflammation, energy metabolism and epigenetic modification (Figure 2). Although there were some literatures to elaborate that m^6^A was related to the occurrence of ovarian aging, more research should be conducted in m^6^A modification on ovarian aging and POI to clarify the pathogenic mechanism to build a complete mechanism-network.

### 3.2 m^6^A and PCOS

PCOS is characterized by ovulation disorder and hyperandrogenemia, which seriously affects women’s reproduction and long-term health, attacking for about 6–10% females (Cooney et al., 2017). Scholars reported in 2012 that IGF-like families (IGF2BP2 and IGFBP2) from cumulus cells in PCOS were abnormal expression (Haouzi et al., 2012). Recently, the authors reported that m^6^A may be involved in the occurrence of PCOS. Zhang et al. analyzed the m^6^A profile of luteinized GCs from normovulatory women and non-obese PCOS patients following controlled ovarian hyperstimulation and found that luteinized GCs of PCOS patients triggered the m^6^A level increased. Meanwhile, the methyltransferases (METTL3/METTL14) and demethylases (FTO and ALKBH5) were also elevated (Figure 2). They identified that m^6^A modification was reduced in FOXO3 mRNA from the luteinized GCs in PCOS patients. Interestingly, selectively knocking down m^6^A methyltransferases or demethylases did not change the expression of FOXO3 in the luteinized GCs of PCOS patients. It indicated that the regulation of FOXO3 by m^6^A modification in PCOS was abnormal (Zhang S. et al., 2020). Moreover, Zhou et al. found that FTO induced the dysfunctions of GCs by upregulating FLOT2, which might be involved in the pathophysiology of obesity PCOS (Zhou L. et al., 2021). In addition, multiple meta-analysis showed that rs9939609 polymorphism of FTO gene was associated with PCOS risk (Wojciechowski et al., 2012; Liu et al., 2017). However, it was still controversial.

## 4 Clinical Application Prospect of m^6^A and its Regulators in Ovarian Diseases

Up to know, the related research of m^6^A modification mainly focused on the basic research, but the related research in clinical transformation were very limited. We summarized potential biomarkers or therapeutic targets of m^6^A regulators related to ovarian diseases. Firstly, m^6^A regulators can be used as biomarkers to reflect gametogenesis disorders, and to achieve preventive treatment of infertility. For example, METTL3, YTHDC1 are possible markers of GV oocyte arrest and GVBD failure (Sui et al., 2020; Mu et al., 2021). KIAA1429 may be the signal of GVBD failure (Hu et al., 2020). Secondly, precise targeting genes such as METTL3 (Sui et al., 2020; Mu et al., 2021), KIAA1429, WTAP (Hu et al., 2020), ALKBH5, YTHDF2, YTHDC1 and YTHDC2 (Ivanova et al., 2017), FTO (Ding et al., 2018; Jiang et al., 2021), may be used as the drugs to ensure normal gametogenesis and exert a better therapeutic effect on oocyte development and improving ovarian function. Finally, inhibitors and activators of m^6^A and its regulators as important treatment strategy have been studied and applied extensively in experimental animals. For instance, the inhibitors of FTO were identified including Rhein (Chen et al., 2012), EGCG (Wu et al., 2018), Entacapone (Peng et al., 2019), Meclofenamic acid (Huang et al., 2015), FB23(Huang Y. et al., 2019), R-2HG (Su et al., 2018), MO-I-500 (Zheng et al., 2014). Gossypolacetic acid (GAA) was reported as the inhibitor of LRPPRC (Zhou et al., 2019). More inhibitors and activators of m^6^A regulators displayed effective therapeutic role in animal disease models. In the future, ovarian related diseases treated with m^6^A regulators might be a promising treatment selection (Figures 1, 2).

## 5 Conclusion

Abnormal m^6^A methylation affected oocyte development and maturation by interfering chromosome/spindle assembly, affecting transcript cutting, translation and degradation, leading to granulosa cell apoptosis and jointly damaging ovarian function. The specific mechanism of m^6^A dynamic regulatory network in the female reproductive system still needs to be further studied. Many unknown areas may be involved in the ovary or oocyte development process, for example, the enhancer and R-loop in the genes modified by m^6^A. In conclusion, m^6^A and its regulators show important function of regulation in ovarian development and oocyte maturation and are potential biomarkers or therapeutic targets for developmental disorders of the oocytes and ovarian diseases.
